# Supplementary material for: Design of a Power Efficient Artificial Neuron Using Superconducting Nanowires
Source: Front Neurosci. 2019 Sep 4;13:933. doi: 10.3389/fnins.2019.00933 (PMC6738026; doi:10.3389/fnins.2019.00933)
Supplement: Supplementary file 1 [file Table_1.DOCX]

**Supplementary Material**

**I. Near-Coincidence Detection**

The neuron may also be under-biased such that two pulses in rapid succession are required to elicit a spike. Figure S1 displays the response of the nanowire neuron to two input pulses with different time delays Δ*t* between them. In this case, in order for the second pulse to fire the neuron, the delay must be less than 3 ns, as shown in Fig. S1b. This behavior is similar to the response of integrating neurons to high frequency inputs [1], and demonstrates that the nanowire neuron may be used for near-coincidence detection of pulses, depending on its biasing conditions.

**
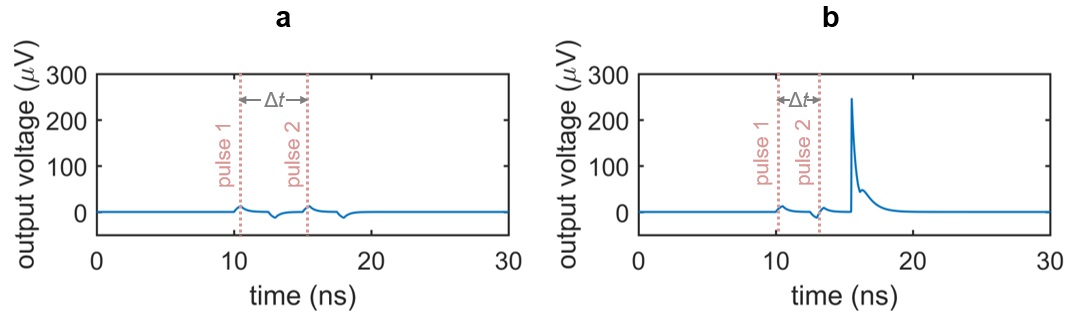
**

**Figure S1 : Near-coincidence detection of pulses.** Output voltage of the neuron when the time between successive input pulses Δ*t* is a) 5 ns b) 3 ns. The pink dashed lines indicate the rising edge of each pulse. Two pulses must be in rapid succession in order to fire the under-biased neuron, demonstrating that it may be used for near-coincidence detection of input pulses. Parameters: *I*_in_ = 4.6 µA, *I*_bias_ = 57.62 µA.

**II. Parabolic Bursting**

Some neurons, such as thalamic and dopaminergic neurons [2], display a unique mode of behavior called bursting, in which the cell alternates between the resting and firing states. The transition between states may be dictated by slow changes in low levels of intracellular calcium ions, which influence the conductance of K+ [3]. As a result, a small, slowly varying signal controls the rapid dynamics of the action potential, leading to alternating periods of resting and firing. This process is considered to be an important aspect of electrical activity in the brain.

        Due to the significance of bursting in biological neurons, past work has sought to replicate similar behavior in platforms such as digital silicon models [4] and Josephson junction models [5] by injecting a low-frequency ac signal into the system. Figure S2 shows the result when a similar technique is applied to the nanowire neuron. In this case, a weak ac signal (*f* = 50 Hz, *I*_ac_ = 4 µA) shown by the dashed red line in Fig. S2a is coupled into the bias port of the neuron, causing the neuron to alternate between the resting and firing states, as reflected in the resulting output voltage signal. A close examination of the timing between adjacent spikes (see Fig. S2b) shows that the spiking frequency increases and then decreases over the firing period, a phenomenon known as parabolic bursting[6]. This behavior was first observed in neuron R15 of the abdominal ganglion of Aplysia [7] [8], and has since been demonstrated in many other cells. The ability of the nanowire neuron to replicate similar dynamics may be therefore be useful for performing a wider range of functions.

**
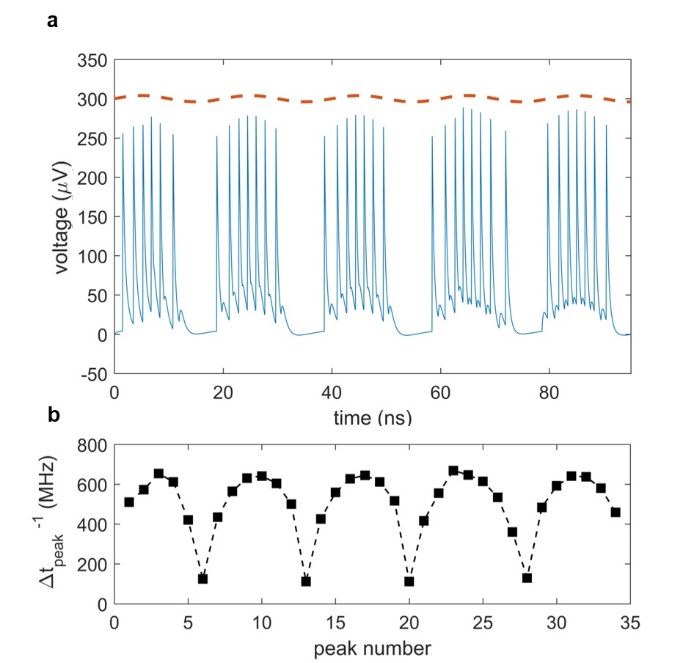
**

**Figure S2: Parabolic bursting in the two-nanowire neuron.** (a) Output voltage of the two-nanowire neuron when the bias is coupled to a weak sinusoidal drive (*f*  = 50 MHz, *I*_ac_ = 4 µA). The red dashed curve indicates the sinusoidal drive, shifted in the y-axis for clarity. (b) The inverse of the time between adjacent peaks shows that the time difference follows a parabolic form. Parameters: *I*_bias_ = 59 µA, *I*_in_ = 6 µA.

**III. Capacitive Synapse**

Although an inductive synapse was chosen for the nanowire neuron due to the advantages of tuning via kinetic inductance, the basic synapse could easily be replaced by a semi-equivalent capacitive model. Figure S3 illustrates an example of a capacitive synapse integrated with the nanowire neuron, and shows both excitatory and inhibitory control.


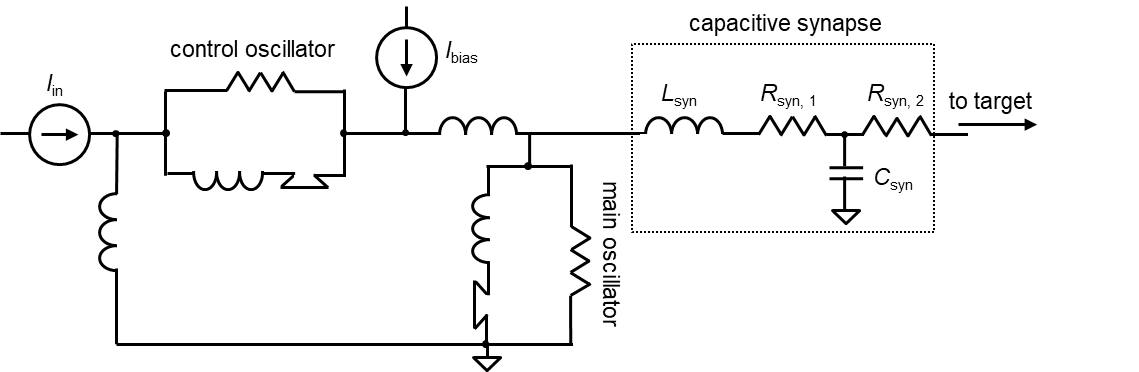


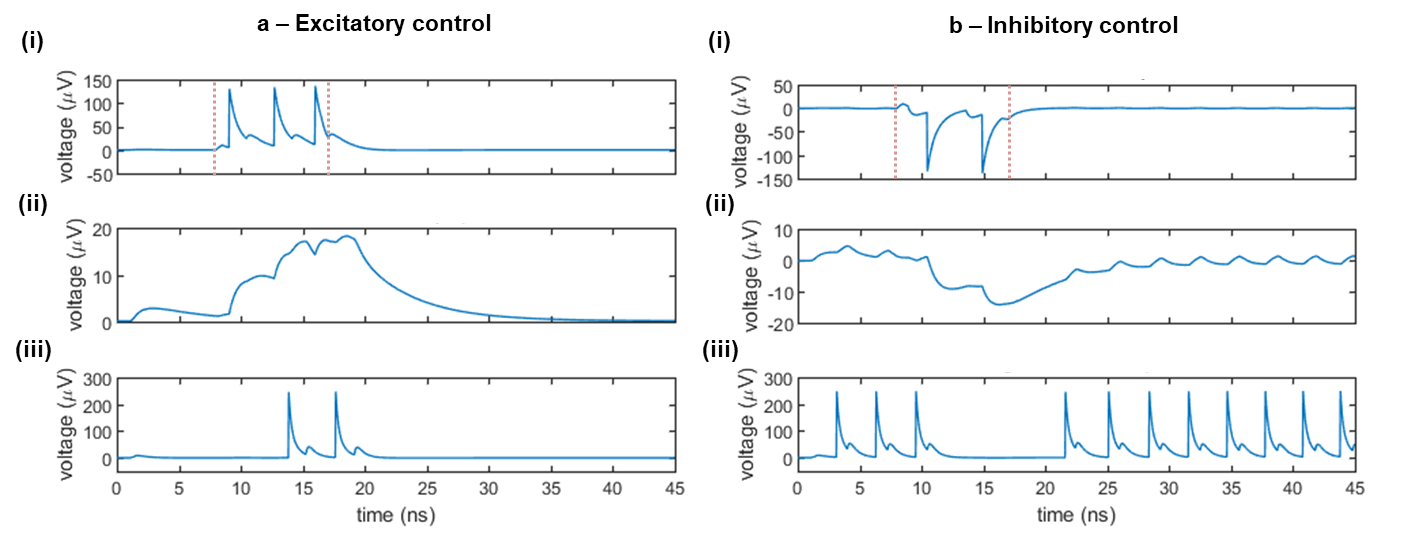


**Figure S3: Capacitive synapse.** (a) Excitatory control. Parameters:  *I*_bias,main_ = 58 µA, *I*_bias,target_ = 56.2 µA, input = 6 µA; *L*_syn_ = 0.1 pH, *C*_syn_ = 1 nF; *R*_syn,1_ = 10 Ω, *R*_syn,2_ = 10 Ω. (i) Output voltage from the first neuron. Dashed red lines indicate the rising edge and falling edge of the input pulse. (ii) Voltage accumulation on the synaptic capacitor. (iii) Output voltage from the target neuron, showing that the neuron only fires in response to the first neuron. (b) Inhibitory control. Parameters: same as in (a), except *I*_bias,main_ = -57.5 µA, *I*_bias,target_ = 57.15 µA. (i) Output voltage from the first neuron. (ii) Voltage accumulated on the capacitor. (iii) Output voltage of the target neuron, showing that the target stops firing in response to the first neuron.

**IV. Tunable Inductive Synapse- Excitatory Control**

The main manuscript showed the effect of modulating the synaptic strength in an inhibitory scenario. However, the modulation is also effective in an excitatory case, as shown in Figure S4. Increasing the modulating current from 0 µA to 8 µA reduces the amount of positive current sent to the target neuron from the synapse. If the current is reduced enough, the target neuron is no longer excited by the main neuron, and stops firing.

*
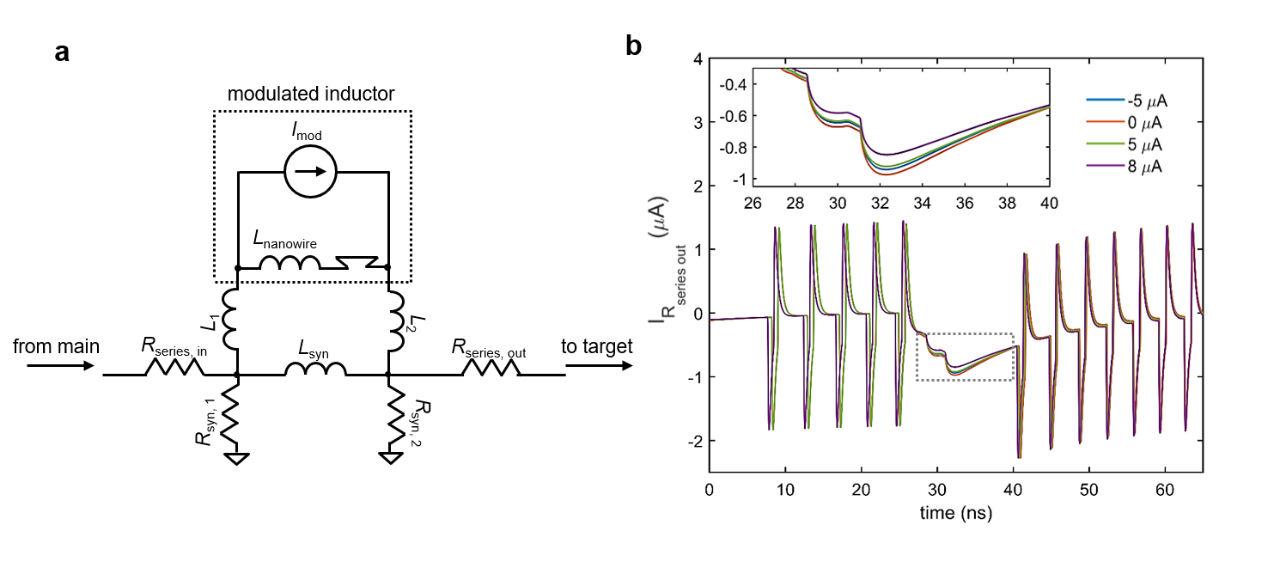
*
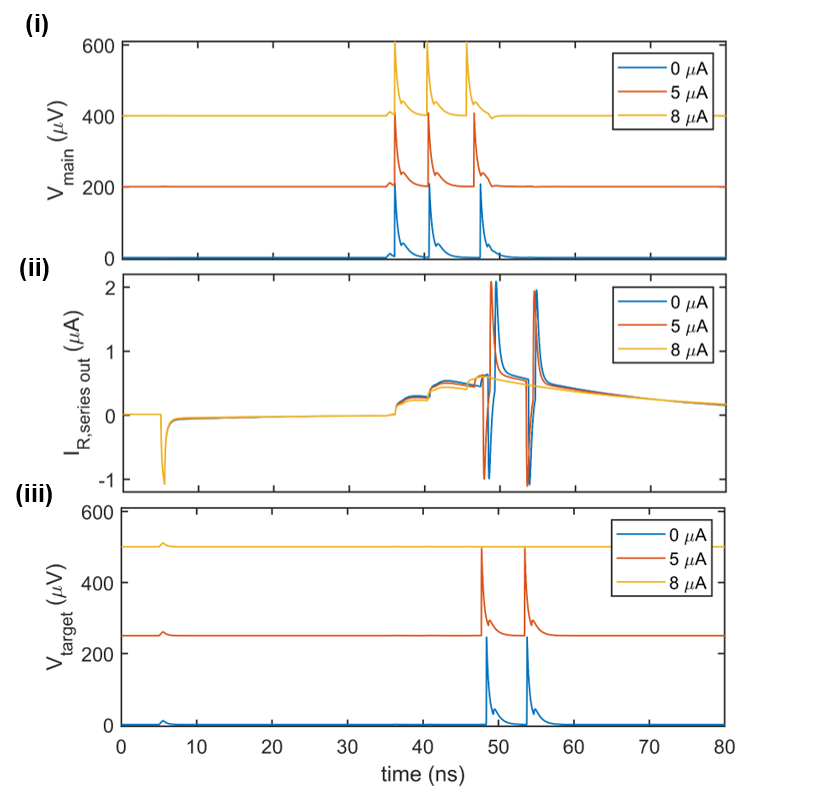


**Figure S4: Example of the tunable inductive synapse with excitatory control.** (i) Voltage output of the main neuron. Traces have been shifted in the y-axis for clarity. The legend indicates the different values of *I*_mod._ (ii) Current through *R*_series,out_ to the target neuron. (iii) Voltage output from the target neuron. Traces have been shifted in the y-axis for clarity. When *I*_mod_ = 8 µA, the synaptic current is too low to excite the target neuron. Parameters: *I*_bias, main_ = 58.1 µA, *I*_bias, target_ = 57.34 µA, *I*_in_ = 4.6 µA, *R*_series,in_ = 12.5 Ω, *R*_syn,1_ = 39 Ω, *R*_syn,2_ = 40 Ω, *R*_series,out_ = 5 Ω, *L*_syn_ = 0.45 µH, *L*_nanowire_ = 0.275 µH, *I*_c, nanowire_ = 6 µA, *L*_1_ = *L*_2_ = 100 pH.

References

[1] E. M. Izhikevich, “Which model to use for cortical spiking neurons?,” *IEEE Trans. Neural Netw.*, vol. 15, no. 5, pp. 1063–1070, Sep. 2004.

[2] R. Bertram, M. J. Butte, T. Kiemel, and A. Sherman, “Topological and phenomenological classification of bursting oscillations,” *Bull. Math. Biol.*, vol. 57, no. 3, pp. 413–439, Jan. 1995.

[3] J. Rinzel, “Bursting oscillations in an excitable membrane model,” in *Ordinary and Partial Differential Equations*, 1985, pp. 304–316.

[4] T. Nanami, K. Aihara, and T. Kohno, “Elliptic and parabolic bursting in a digital silicon neuron model,” presented at the 2016 International Symposium on Nonlinear Theory and Its Applications, Yugawara, Japan, 2016, p. 4.

[5] S. K. Dana, D. C. Sengupta, and C.- Hu, “Spiking and Bursting in Josephson Junction,” *IEEE Trans. Circuits Syst. II Express Briefs*, vol. 53, no. 10, pp. 1031–1034, Oct. 2006.

[6] C. Soto-Treviño, N. Kopell, and D. Watson, “Parabolic bursting revisited,” *J. Math. Biol.*, vol. 35, no. 1, pp. 114–128, Nov. 1996.

[7] F. Strumwasser, “The demonstration and manipulation of a circadian rhythm in a single neuron,” in *Circadian clocks*, Amsterdam: North-Holland Publishing Co., 1965, pp. 442–462.

[8] B. O. Alving, “Spontaneous Activity in Isolated Somata of Aplysia Pacemaker Neurons,” *J. Gen. Physiol.*, vol. 51, no. 1, p. 29, Jan. 1968.
